# New Insights on *Euphorbia dendroides* L. (*Euphorbiaceae*): Polyphenol Profile and Biological Properties of Hydroalcoholic Extracts from Aerial Parts

**DOI:** 10.3390/plants10081621

**Published:** 2021-08-06

**Authors:** Antonella Smeriglio, Marcella Denaro, Domenico Trombetta, Salvatore Ragusa, Clara Circosta

**Affiliations:** 1 Department of Chemical, Biological, Pharmaceutical and Environmental Sciences, University of Messina, Viale Ferdinando Stagno d’Alcontres 31, 98166 Messina, Italy; marcella.denaro@unime.it (M.D.); domenico.trombetta@unime.it (D.T.); clara.circosta@unime.it (C.C.); 2 Department of Health Sciences, University “Magna Graecia” of Catanzaro, Viale Europa, 88100 Catanzaro, Italy; srgusa@unicz.it

**Keywords:** *Euphorbia dendroides* L., aerial parts, polyphenols, antioxidant activity, anti-inflammatory activity, toxicity

## Abstract

*Euphorbia dendroides* L. is a rounded shrub commonly found in the Mediterranean area well-known, since ancient times, for its traditional use. The aim of the present study was to investigate the phytochemical profile as well as the antioxidant and anti-inflammatory properties of flower (FE), leaf (LE), fruit (FrE), and branch (BE) hydroalcoholic extracts. For this purpose, a preliminary phytochemical screening followed by RP-LC-DAD-ESI-MS analysis, as well as several in vitro cell-free colorimetric assays, were carried out. Moreover, the toxicity of the extracts was investigated by the brine shrimp lethality assay. All extracts showed a high content of polyphenols, in particular phenolic acids (chlorogenic acid 0.74–13.80 g/100 g) and flavonoids (rutin 0.05–2.76 g/100 g and isovitexin 8.02 in BE). All the extracts showed strong and concentration-dependent antioxidant and anti-inflammatory activity with, on average, the following order of potency: FE, LE, FrE, and BE. Interestingly, all the extracts investigated did not show any toxicity on *Artemia salina*. Moreover, the only LD_50_ found (BE, 8.82 mg/mL) is well above the concentration range, which has been shown the biological properties. Considering this, this study offers the first evidence of the possible use of the polyphenol extracts from the aerial parts of *E. dendroides* as promising antioxidant and anti-inflammatory agents.

## 1. Introduction

*Euphorbia* (Euphorbiaceae) is the third most common genus among flowering plants [1]. It is widespread around the world with more than 2000 species and with an exceptional diversity such as shrubs, vines, and grassy plants [1]. *Euphorbia* is well known, since ancient times, for its therapeutic activity on several gastrointestinal ailments, infections, skin irritations, body pain, microbial illness, sensory disorders, and as an antidote against snake venom [2,3,4]. Numerous species are commonly cultivated for ornamental purposes, such as *E. pulcherrima* Willd., *E. fulgens* Karw, *E. milii* Des Moul., *E. milii* var. *splendens* Ursch and Leandri, *E. tirucalli* L., and *E. lactea* Roxb [5].

Moreover, *E. pekinensis* Rupr., *E. kansui* Liou, *E. lathyris* L., *E. humifusa* Willd., and *E. maculata* L. were described by the Chinese Pharmacopoeia for their application in traditional medicine against gonorrhea, migraine, edema, and warts [6].

All plants belonging to the *Euphorbia* genus are characterized by the presence of an irritating latex, which plays a pivotal role in the first defense mechanism against insects, pathogens, and herbivores. This latex is a rich source of phytochemicals, which have been extensively investigated over time [7,8,9].

More than 650 types of diterpenes and triterpenes with several biological properties such as antiproliferative, antimicrobial, antiviral, anti-inflammatory, vasoactive, cytotoxic, neuroprotective, and anticancer, have been identified in the *Euphorbia* genus, thus, supporting the traditional use of the *Euphorbia* spp. [4,7]. Ingenol mebutate, a diterpene isolated from *E. peplus* L., showed significant activity against the early stage of actinic keratosis following topical application [10]. These promising biological activities led to an increased interest in *Euphorbia* spp. [4,8,11]. However, despite this, there are many under-investigated species, such as *E. dendroides* L., characterized by deciduous leaves, for which only limited information is available today. Moreover, polyphenols, are under-looked compounds in this plant genus.

*E. dendroides* is a rounded shrub or a small tree, commonly found in Mediterranean areas (Spain, France, Italy, Balkans, Greece, Turkey, Northern Africa, etc.) along coasts and especially on rocks, cliffs, and arid calcareous soils. The few studies available are mainly focused on its latex with particular reference to terpenoids [12,13,14,15,16], while to the authors’ knowledge, only one study is currently available on extracts obtained from the whole plant, which investigated the antioxidant and anti-proliferative activity of this species [17].

In our previous study, the phytochemical profile as well as the antioxidant and anti-acetylcholinesterase activities of *E. dendroides* latex, were investigated [9]. The latex extract showed interesting biological properties without any significant toxicity on *Artemia salina,* paving the way for new potential uses of *E. dendroides* latex for example as a safe, effective, and environmentally friendly insecticide [9].

Considering this, and given the lack of studies concerning the polyphenol extracts of this plant, we decided to focus our attention on the aerial parts (leaf, branch, flower, and fruit), with the aim to extend the knowledge about the polyphenol profile as well as on the antioxidant and anti-inflammatory properties of this plant species, highlighting also its potential ecotoxicity.

## 2. Results

**Figure 1:**
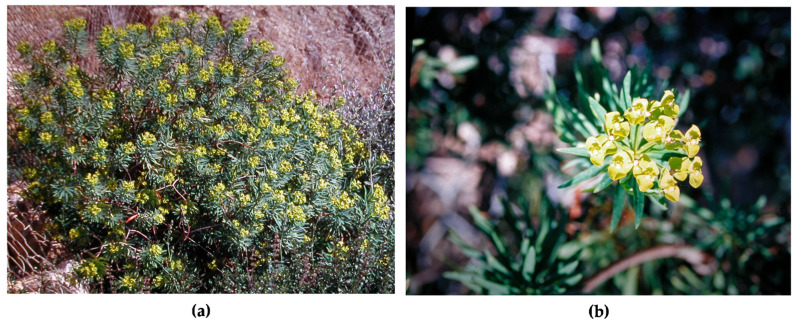
*Euphorbia dendroides* L. (**a**) and particular of the aerial parts (**b**). Original photo by SR.

In the present study, the phytochemical profile as well as the antioxidant, anti-inflammatory, and toxicity of leaf (LE), branch (BE), flower (FE), and fruit (FrE) hydroalcoholic extracts of *E. dendroides* L. (Figure 1) were evaluated for the first time. 

The extraction method adopted has allowed the obtainment of high extraction yields ranging from 13.70 to 24.21%, with flowers, which showed the best extraction yield, followed by leaves, branches, and fruits. 

### 2.1. Phytochemical Screening and Characterization of Polyphenol Profile

Table 1 shows the results of the preliminary phytochemical screening carried out by several in vitro colorimetric assays. 

All extracts, albeit with substantial differences, showed a high content of polyphenols. FE showed the highest content of total phenols expressed as g of gallic acid equivalents (GAE)/100 g of dry extract (DE) followed by LE, FrE, and BE. Comparable amounts of flavonoids, expressed as g of quercetin equivalents (QE)/100 g DE, were detected in LE and FrE, followed by FE. On the contrary, a consistent decrease of flavonoids content was observed in BE. The vanillin index and proanthocyanin assays were carried out in order to evaluate the flavonols and proanthocyanidins content. Moreover, these two assays, in particular their ratio (vanillin index/proanthocyanidins), allow the calculation of the polymerization degree. All extracts, which showed the highest proanthocyanidins content with respect to the flavonols content, showed a low polymerization degree (≤1). This allows for postulating the high presence of monomeric molecules.

**Figure 2:**
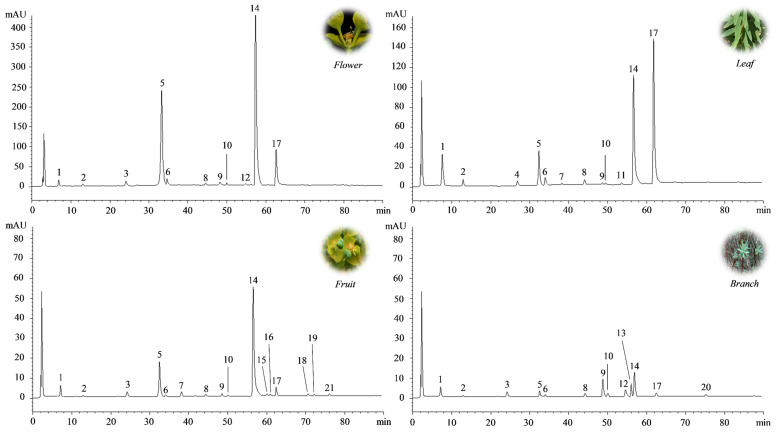
Representative LC-DAD chromatograms of *E. dendroides* flower (FE), leaf (LE), fruit (FrE), and branch (BE) extracts acquired at 260 nm. Peak numbers refer to compounds listed in Table 2.

These preliminary results were confirmed by the determination of the polyphenol profile by reverse-phase liquid chromatography coupled with diode array detection and electrospray ion trap mass spectrometry (RP-LC-DAD-ESI-MS) analysis (Figure 2), which identified the FE as the richest source of polyphenols (17.23 g/100 g DE), followed by LE, FrE, and BE (Table 2).

More than 10 compounds were identified and quantified in each extract by RP-LC-DAD-ESI-MS analysis (Figure 1; Table 2). Among them, a prevalence of chlorogenic acid and rutin was recorded in all the extracts investigated, with the exception of BE, in which the most abundant compound was isovitexin (8.02 g/100 g DE). However, the amount of both compounds changed significantly depending on the aerial part investigated. Indeed, chlorogenic acid, although it is more abundant in FE (13.80 g/100 g DE), is still expressed at high levels and in comparable amounts to RE and LE. On the contrary, the rutin content, which is always higher in FE (2.76 g/100 g DE), decreases significantly (~4 fold) in the other two extracts (RE and LE). A completely different polyphenol profile was discovered in BE, where chlorogenic acid content is clearly lower than other extracts (0.74 g/100 g DE) and rutin amount (0.05 g/100 g DE) is comparable to other flavonoids identified. Here, the most abundant compound was the isovitexin, a C-glycosylated flavonoid, generally abundant in the plant cortex. 

### 2.2. Antioxidant and Anti-Inflammatory Activity

**Figure 3:**
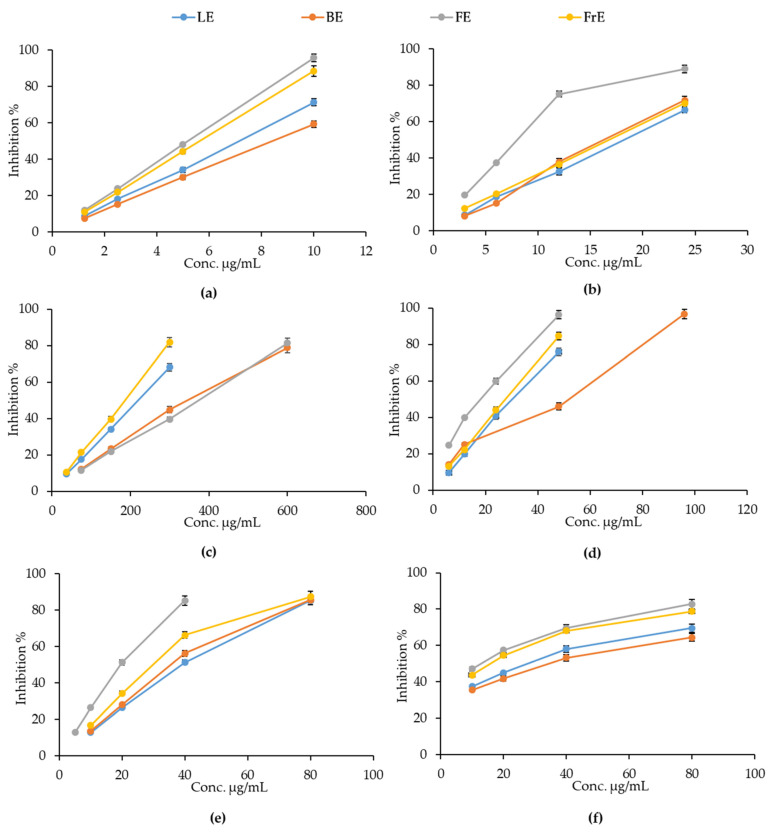
The antioxidant and free radical-scavenging activity of *E. dendroides* flower (FE), leaf (LE), fruit (FrE), and branch (BE) extracts towards ORAC (**a**); TEAC (**b**); Iron chelating-activity (**c**); FRAP (**d**); DPPH (**e**) and β-carotene bleaching (**f**) assay. Results were expressed as the mean inhibition percentage (%) ± standard deviation of three independent experiments (*n* = 3).

The antioxidant and free radical-scavenging activity of the *E. dendroides* extracts was evaluated by several cell-free colorimetric assays, based on different environments and reaction mechanisms (hydrogen atom transfer and electron transfer-based methods). As depicted in Figure 3, all extracts showed a concentration-dependent antioxidant and free radical-scavenging activity in each test performed. 

However, the response of the extracts changes a lot depending on the aerial part considered. The oxygen radical absorbance capacity (ORAC) was identified as the main mechanism through which all the extracts exert their antioxidant activity, as can be observed from the lowest half-maximal inhibitory concentrations (IC_50_) reported in Table 3. However, in this case, extracts did not show any statistically significant differences.

FE showed the most powerful antioxidant and free-radical scavenging activity in all assays carried out, with the exception of the iron-chelating activity, in which RE showed the best and statistically significant antioxidant activity with respect to all other *E. dendroides* extracts. In particular, statistically significant results, with respect to LE, FrE, and BE, were observed for Trolox equivalent antioxidant capacity (TEAC) and DPPH assays. On the contrary, FE showed statistically significant results with respect to LE, and LE, and FrE in ferric reducing antioxidant power (FRAP) and β-carotene bleaching (BCB) assays, respectively. All extracts showed statistically significant results with respect to Trolox, ethylenediaminetetraacetic acid (EDTA), and butylhydroxytoluene (BHT), used as reference compounds.

**Figure 4:**
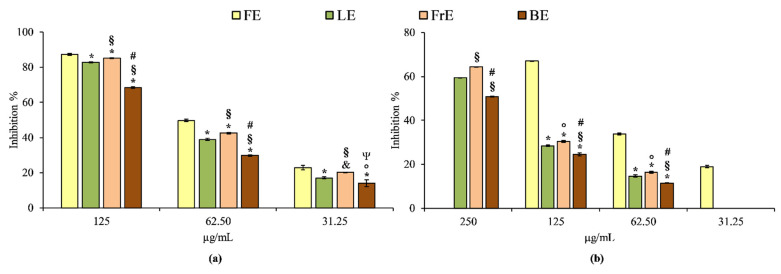
The anti-inflammatory activity of *E. dendroides* flower (FE), leaf (LE), fruit (FrE) and branch (BE) extracts towards BSA denaturation assay (**a**) and protease inhibition assay (**b**). Results were expressed as the mean inhibition percentage (%) ± standard deviation of three independent experiments (*n* = 3). * *p* < 0.001 vs. FE; ^§^ *p* < 0.001 vs. LE; ^#^ *p* < 0.001 vs. FrE; ^&^ *p* < 0.05; ^°^ *p* < 0.05 vs. LE; ^Ψ^ *p* < 0.05 vs. FrE.

The anti-inflammatory properties of *E. dendroides* extracts were evaluated by two simple in vitro cell-free based assays, which evaluate the heat-induced protein denaturation (BSA denaturation assay) and the protease inhibitory activity. These assays allow simultaneous evaluation of enzymatic and non-enzymatic anti-inflammatory mechanisms. The results obtained showed that, first of all, the extracts show once again a concentration-dependent behavior, in accordance with what has already been seen in the antioxidant activity (Figure 4).

According to the antioxidant assays, FE is also the strongest anti-inflammatory agent among the extracts investigated both in BSA denaturation and protease inhibition assay. The order of potency, expressed as IC_50_ is the following: 62.88 µg/mL (C.L. 60.55–64.34 µg/mL) for FE followed by LE (IC_50_ 80.27 µg/mL; C.L. 78.48–82.66 µg/mL), FrE (IC_50_ 73.50 µg/mL; C.L.72.22–74.84 µg/mL) and BE (IC_50_ 104.80 µg/mL; C.L. 103.55–106.15 µg/mL) in the BSA denaturation assay, and 92.27 µg/mL (C.L. 90.45–94.05 µg/mL) for FE followed by FrE (IC_50_ 205.34 µg/mL; C.L. 203.89–207.23 µg/mL), LE (IC_50_ 219.02 µg/mL; C.L. 216.56–221.29 µg/mL) and BE (IC_50_ 253.84 µg/mL; C.L. 250.45–257.59 µg/mL) in the protease inhibition test. Diclofenac sodium, used as a reference compound in both assays, showed the following IC_50_ values: 42.37 µg/mL (C.L. 40.55–44.68) and 36.55 µg/mL (C.L. 34.88–38.79) in BSA denaturation and protease inhibition assay, respectively. Considering this, all *E. dendroides* extracts showed a different and statistically significant (*p* < 0.001) anti-inflammatory behavior in all tests carried out, both among them and with respect to the reference compound diclofenac sodium.

### 2.3. Brine Shirmp Letality Assay

The brine shrimp lethality assay is a useful and rapid screening tool to evaluate the toxicity of plant complexes or pure compounds [18]. *E. dendroides* extracts were tested at different concentrations ranging from 0.0001 to 10 mg/mL. No mortality was found at 24 h, while only weak toxicity (ranging from 0 to 10%, data not shown) was found after 48 h and only at the highest concentration (10 mg/mL) for FE, LE, and FrE. Despite all extracts not showing any significant toxicity over a broad range of concentrations, BE proved to be the most toxic extract among those investigated affecting the larvae viability with an LD_50_ value of 8.82 mg/mL (C.L. 7.05–9.16 mg/ml).

## 3. Discussion

The *Euphorbia* species are plants well known for their health effects and traditional use around the world [5]. Their biological activity is closely related to the phytochemical profile which, however, may vary according to different parameters such as species, grown conditions, the part of the plant investigated, as well as applied extraction methods. 

Much research has been carried out in order to highlight the phytochemical profile of different *Euphorbia* spp. This has allowed the identification of numerous classes of secondary metabolites with marked health properties, including multi-drug resistance modulatory activity, antiproliferative, cytotoxic, antiviral, antimicrobial, antioxidant, and anti-inflammatory activity [5,9,13,14,17,19,20]. 

Among these compounds, the most representative are sesquiterpenes, diterpenes, triterpenes, steroids, cerebrosides, phenolic compounds, tannins, and coumarins [5,20]. 

In particular, it is well known that the *Euphorbia* genus is a rich source of jatrophane and modified jatrophane diterpenes, a wide range of structurally unique macrocyclic and polyoxygenated derivatives, which opened new frontiers for research studies on this genus [21]. Many of them have been isolated from the latex and aerial parts of E. *dendroides* and, for this reason, the phytochemical research on this species has mainly focused in recent years on this class of compounds [12,13,15,19].

To date, although polyphenols are among the most investigated compounds in the plant kingdom, studies concerning these secondary metabolites in the *Euphorbia* genus are rather scarce and even missing for some species.

In this study, the polyphenols profile of hydroalcoholic extracts of aerial parts (flower, leaf, fruit, and branch) of *E. dendroides* was investigated for the first time. Moreover, to date and to the authors’ knowledge, only one study, which investigated the antiproliferative and antioxidant activities of two fractions (ethyl acetate and butanol) of whole plant methanol/water extract of *E. dendroides* is currently available [17]. 

The extraction method adopted in the present study allowed to obtain a very high extraction yield (13.70–24.21%) with respect to previous investigations (0.42–2.12%) [17]. Despite the polyphenols extraction from plant material is affected by several parameters such as chemical environment, the extraction process, and solvent polarity, as well as particle size and storage conditions [22], the results of the present study are in accordance with Ghout et al. [17] showing a total phenols content ranging from 26.54 to 64.75 g GAE/100 g DE with respect to 16.43 and 92.95 g GAE/100 g DE for butanol and ethyl acetate fractions of *E. dendroides* extract. 

On the contrary, a greater content of flavonoids was found in the hydroalcoholic extract of flowers, leaves, fruits, and branches analysed in this study compared to the flavonoid content found in the two fractions mentioned above (46.93–84.85 g QE/100 g DE vs. 1.22 and 2.60 g QE/100 g DE) [17]. Moreover, extracts from aerial parts of *E. dendroides* showed a greater polyphenols content in comparison with the methanol latex extract, which showed a total phenols and flavonoids content equal to 4.75 g GAE/100 g and 1.47 g RE/100 g DE, respectively [9].

In the present study, the RP-LC-DAD-ESI-MS analysis allowed us to identify and quantify more than 10 compounds in each extract, according to previous results which identify ten [17] and fourteen compounds [9] in different *E. dendroides* extracts. According to Ghout et al. [17], chlorogenic acid is the most representative phenolic acid in all the extracts here investigated with the exception of the BE, in which the most abundant compound was the flavonoid isovitexin. However, expressing the results of Ghout et al. [17] as dry weight, the chlorogenic acid content found in FE, LE and FrE was about double. On the contrary, although it remains the second most abundant phenolic acid present in the extracts under consideration, the gallic acid content is about 100 times lower in the extracts analysed in the present study compared to those analysed previously [17]. Caffeic acid represent the second most abundant phenolic acid in LE, whereas comparable amount was found in the other extracts. This phenolic acid was identified also previously in the methanol latex extract [9] as well as in the methanol/water extract of whole plant [17]. Finally, according to previous results, other minor phenolic acids such as *p*-cumaric acid and *p*-hydroxybenzoic acid [9,17] were found. Beyond the phenolic acids, the extracts investigated in the present study showed, unlike those investigated previously, a high content of flavonoids as well, with rutin as the most abundant compound ranging from 200 to10,000 times higher with respect to previous results [17]. This flavonoid was identified also in other *Euphorbia* species such as *E. lathyris* and *E. geniculate*, in which also other quercetin derivatives such as quercetin-3-*O*-rhamnoside and quercetin-3-*O*-D-glucopyranoside as well as the aglycon (quercetin) were identified [20]. On the contrary, rutin is completely absent in the methanol latex extract previously investigated, characterized by the abundant presence of eryodictiol-7-*O*-glucoside, eriodyctiol, naringenin-7-*O*-glucoside, naringenin, and quercetin [9]. 

Two biological activities (antioxidant and anti-inflammatory) were investigated in the present study. According to previous results, all *E. dendroides* extracts showed concentration-dependent antioxidant [9,17] and anti-inflammatory activity. 

The different in vitro colorimetric assays carried out, characterized by different environment and reaction mechanisms, allowed identification of the oxygen radical absorbance capacity as the main mechanism through which all the extracts exert their antioxidant activity. Despite all the analysed extracts showing significantly lower antioxidant activity compared to the reference standards, in accordance with what was previously observed, the IC_50_ values found in the present study are much lower than those previously highlighted in the literature for *E. dendroides* extracts [9,17], showing the most powerful antioxidant activity in the aerial part extracts.

On average, the extracts proved to be particularly active in tests based on hydrogen atoms-transfer reactions (ORAC and BCB), followed by electron and hydrogen atom-transfer-based assays (TEAC and DPPH), and finally on tests based on electrons transfer (FRAP). The iron-chelating activity was the lowest among those investigated, although the FrE showed interesting results. It is well-known that several flavonoids efficiently chelate trace metals such as iron and copper, which play an important role in oxygen metabolism avoiding the generation of highly aggressive secondary radical species [23]. The proposed binding sites for trace metals to flavonoids in order of potency are the following: catechol moiety in B ring, 3-hydroxyl, 4-oxo groups in the heterocyclic ring, and the 4-oxo, 5-hydroxyl groups between the heterocyclic and the A rings [23]. Considering this, certainly, rutin exerts the greatest contribution in terms of iron-chelating activity, although the presence of different flavonoids could enhance this activity. It is well-known that the antioxidant behaviour of plant extracts is strictly related to the quali-quantitative composition of the polyphenolic profile. In particular, the reducing ability depends on the number of free-hydroxyl groups on the base skeleton [9,24]. Considering this, among phenolic acids, chlorogenic acid plays a predominant role followed by gallic, caffeic, and dihydroxybenzoic acid. On the contrary, for flavonoids, the radical scavenging activity depends on the structure and the substituents of the heterocyclic and B rings and, in particular, by the catechol group in the B ring, which has the better electron-donating properties. Moreover, the 2,3-double bond conjugated with the 4-oxo group is responsible for electron delocalization. The presence of a 3-hydroxyl group in the heterocyclic ring also increases the radical-scavenging activity, while additional hydroxyl or methoxy groups at positions 3,5, and 7 of A and C rings seem to be less important [23]. Considering this, the order of potency of flavonoids is the following: flavonols, flavones, flavanols, and flavanones. Most of the isolated flavonoids from the *Euphorbia* genus are simple flavonols as well as O-, C-substituted and prenylated. The main glycosides are d-glucose, l-rhamnose, or glucorhamnose attached at either the C-3 or C-7 position. Structure–activity relationship studies showed that methylation of the hydroxyl groups on the C-3 or C-7 position decreases the activities while glycosylation loses the activity. In any case, the parent compound is essential in preserving biological activity [20]. Indeed, observing the average behaviour of the extracts in terms of antioxidant activity, it can be deduced that FE, the extract richer in rutin and isorhamnetin-3-*O*-rutinoside (both flavonols), shows the strongest antioxidant activity, followed by FrE, which is the most diversified extract in terms of flavonoid content, followed by LE and BE. BE in particular, shows the lowest antioxidant activity probably attributable to the very low concentration of rutin found compared to the other extracts analysed and to the high presence of isovitexin, which, as a C-glycosylated flavonoid, acts as a weak antioxidant [23].

The present study showed that *E. dendroides* extracts from aerial parts showed also strong and concentration-dependent anti-inflammatory properties both in enzymatic and non-enzymatic assays. It is well-known that polyphenols may exert anti-inflammatory effects through various mechanisms. Among the main ones that can be counted there is certainly the radical scavenging activity, due to the close connection between oxidative balance and inflammation, followed by modulation of the main enzymes involved in inflammation such as protease, COX-1 and COX-2, and phospholipase A2, as well as through complex signalling pathways that lead to a modulation of the release of important pro-inflammatory markers such as interleukins and nitric oxide [25].

One of the most important aspects of this study is that the extracts under examination showed such biological activities without showing any toxic effect on *Artemia salina*, as demonstrated previously for other leaf and flowering top extracts [26].

Moreover, this study demonstrates indirectly that the toxicity generally associated with *E. dendroides* is mainly due to the terpenes fraction in accordance with what was observed in our previous study on a methanol extract of *E. dendroides* latex, in which an LD_50_ ~350 times lower with respect to the most toxic extract investigated in the present study (0.025 mg/mL vs. 8.82 mg/mL) was found [9].

## 4. Materials and Methods

### 4.1. Chemicals

The 1,1-Diphenyl-2-picrylhydrazyl radical (DPPH), 2,20-azino-bis(3-ethylbenzothiazoline-6-sulfonic acid (ABTS), potassium persulfate (K_2_S_2_O_8_), butylate-hydroxytoluene, phenazine methosulphate, 6-Hydroxy-2,5,7,8-tetramethylchromane-2-carboxylic acid (Trolox), diclofenac sodium, butylated hydroxytoluene (BHT), 2,2′-Azobis(2-methylpropionamidine) dihydrochloride (AAPH), fluorescein sodium salt, sodium phosphate dibasic (Na_2_HPO_4_), potassium phosphate monobasic (KH_2_PO_4_), 2,4,6-Tris(2-pyridyl)-S-triazine (TPTZ), iron sulphate heptahydrate, sodium acetate, sodium carbonate, vanillin, Folin-Ciocalteu reagent, β-carotene, linoleic acid, tween 80, ferrozine, iron (II) chloride, EDTA, fatty free BSA, trypsin, tris-HCl, casein, potassium bichromate (K_2_Cr_2_O_7_) were purchased from Sigma-Aldrich (MSt. Louis, MO, USA). Methanol, acetonitrile, glacial acetic acid and phosphoric acid were HPLC grade and purchased from Merck (Darmstadt, Germany). Reference compounds were HPLC grade and purchased from Extrasynthese (Genay, France).

### 4.2. Plant Material Collection and Samples Preparation

*Euphorbia dendroides* L. (Euphorbiaceae) was collected and botanically identified by Prof. S. Ragusa in April 2019 in Messina (Italy), Masse locality (200 m a.s.l.) during flowering and fructification. A voucher specimen (19/04 ED) has been deposited within the herbarium of the Department ChiBioFarAm, University of Messina, Messina, Italy. 

The aerial parts have been manually divided into leaves, flowers, fruits, and branches, that have been air-dried in the dark at RT and then powdered by a blade mill (IKA^®^ A11) with liquid nitrogen in order to block enzymatic activities and preserve the chemical features. Two grams of each frozen powder (leaves, flowers, fruits, and branches) were extracted with 40 mL of a hydroalcoholic mixture consisting of EtOH/H_2_O 80:20 *v*/*v*, vortex-mixed for 3 min, and sonicated in an ice-cold bath for 5 min using a 3 mm titanium probe set at 200 W and 30% amplitude signal (Vibra Cell™ Sonics Materials, inc., Danbury, CT, USA). Extracts were then centrifuged at 3000 × g for 10 min. Thereafter, the supernatant was filtered on filter paper and evaporated to dryness by a rotary evaporator at 37 °C. The procedure was repeated 3 times. Dry extracts were suspended and properly diluted in a hydroalcoholic mixture for phytochemical characterization and subsequent analyses. 

### 4.3. Phytochemical Screening

#### 4.3.1. Total Phenols Content

Total phenols content was determined using the Folin–Ciocalteu method according to Smeriglio et al. [27]. Briefly, 50 µL of sample solution (0.5–4.0 mg/mL) and 450 µL of deionized water were added to 500 µL of Folin–Ciocalteu reagent. After 3 min, sodium carbonate (500 µL, 10% *v*/*v*) was added and samples were left in the dark at RT for 1 hour, vortexing every 10 min. Absorbance was read at 785 nm with a UV-VIS spectrophotometer (Model UV-1601, Shimadzu, Kyoto, Japan) against a blank consisting of the same extraction hydroalcoholic mixture. Gallic acid was used as a reference compound (0.075–0.60 µg/mL). Results, which represent the average of three independent experiments in triplicate (*n* = 3) were expressed as g GAE/100 g DE. 

#### 4.3.2. Total Flavonoids Content

Total flavonoids content was determined as reported by Smeriglio et al. [9]. Briefly, 0.2 mL of sample solution (0.5–4.0 mg/mL) were mixed with 0.2 mL of AlCl_3_ (2 mg/mL) and 1.2 mL of sodium acetate (50 mg/mL). After 2.5 h, the absorbance was recorded at 440 nm with a UV-VIS spectrophotometer (Model UV-1601, Shimadzu, Kyoto, Japan) against a blank consisting of the same extraction hydroalcoholic mixture. Quercetin was used as a reference compound (0.125–1.0 mg/mL). Results, which represent the average of three independent experiments in triplicate (*n* = 3), were expressed as g QE/100 g DE.

#### 4.3.3. Vanillin Index Determination

Vanillin index was evaluated according to Smeriglio et al. [24]. Briefly, 2.0 mL of sample solution diluted in 0.5 M H_2_SO_4_ (absorbance ranging from 0.2 to 0.4) were loaded onto a conditioned Sep-Pak C18 cartridge (Waters, Milan, Italy). The column was washed with 2.0 ml of H_2_SO_4_ (5.0 mM), purged with air, and charged with methanol (5.0 mL) to elute the sample. Thereafter, 1 mL of the eluate was added to 6.0 mL of 4% vanillin methanol solution, and the mixture was conditioned in a water bath at 20 °C for 10 min. Chloridric acid (3.0 ml) was added and after incubation time (15 min), the absorbance was recorded at 500 nm with a UV-VIS spectrophotometer (Model UV-1601, Shimadzu, Kyoto, Japan) against a blank consisting of the same extraction hydroalcoholic mixture. Catechin was used as a reference compound (0.125–0.50 mg/mL). Results, which represent the average of three independent experiments in triplicate (*n* = 3), were expressed as g CE/100 g DE. 

#### 4.3.4. Proanthocyanidins Determination

The proanthocyanidins content was evaluated as described by Barreca et al. [28]. Briefly, 2.0 mL of sample solution, diluted 10 times with 0.05 M H_2_SO_4,_ was loaded onto a conditioned Sep-Pak C18 cartridge (Waters, Milan, Italy), preconditioned with 5 mM H_2_SO_4_ (2.0 mL), and purged with air. The proanthocyanidins-reach fraction obtained was eluted with methanol (3.0 mL) and collected in a 100 ml flask shielded from light containing 9.5 mL of absolute ethanol. Thereafter, the mixture was added with 12.5 mL of FeSO_4_ · 7H_2_O solubilized in 37% HCl (300 mg/L) and placed to reflux for 50 min. After cooling by immersion in cold water (20 °C) for ten min, the absorbance was read at 550 nm with an UV-VIS spectrophotometer (Model UV-1601, Shimadzu, Kyoto, Japan) against a blank consisting of the same extraction hydroalcoholic mixture. The basal anthocyanins content of the sample was determined detracting the absorbance of a sample prepared under the same conditions but simply cooled in ice. Proanthocyanidins content was expressed as 5 times the amount of cyanidin formed by means of a cyanidin chloride (ε = 34,700) calibration curve. Results, which represent the average of three independent experiments in triplicate (*n* = 3), were expressed as g CyE/100 g DE.

### 4.4. Determination of Polyphenol Profile by RP-LC-DAD-ESI-MS Analysis

Polyphenols characterization of LE, FE, FrE, and BE was carried out according to Smeriglio et al. [29] by RP-LC-DAD-ESI-MS analysis. Separation was carried out by a Luna Omega PS C18 column (150 mm × 2.1 mm, 5 μm; Phenomenex, Torrance, CA, United States) at 25 °C by using a mobile phase consisting of solvent A (0.1% formic acid) and solvent B (methanol) according to the following elution program: 0–3 min, 0% B; 3–9 min, 3% B; 9–24 min, 12% B; 24–30 min, 20% B; 30–33 min, 20% B; 33–43 min, 30% B; 43–63 min, 50% B; 63–66 min, 50% B; 66–76 min, 60% B; 76–81 min, 60% B; 81–86 min, 0% B and equilibrated 4 min for a total run time of 90 min. The injection volume was 5 µL. The UV-Vis spectra were recorded ranging from 190 to 600 nm and chromatograms were acquired at different wavelengths (260, 292, 330, and 370 nm) to identified all polyphenol classes. The experimental parameters of the mass spectrometer (ion trap, model 6320, Agilent Technologies, Santa Clara, CA, USA) operating in negative (ESI−) ionization mode were set as follows: capillary voltage 3.5 kV, nebulizer (N_2_) pressure 40 psi, drying gas temperature 350 °C, drying gas flow 9 L/min and skimmer voltage 40 V. Acquisition was carried out in full-scan mode (90–1000 *m*/*z*). Data were acquired by Agilent ChemStation software version B.01.03 and Agilent trap control software version 6.2.

### 4.5. Antioxidant and Free-Radical Scavenging Activity

*The antioxidant* activity of *E. dendroides* extracts was evaluated by several in vitro colorimetric assays based on different mechanisms (electron, hydrogen and electron, and hydrogen transfer-based assays) and reaction environments. Absorbance was recorded by a UV-VIS spectrophotometer (Model UV-1601, Shimadzu, Kyoto, Japan). Results, which represent the average of three independent experiments in triplicate (*n* = 3), were expressed as inhibition percentage (%) of the oxidative/radical activity, calculating the IC_50_ with the respective C.L. at 95% by Litchfield and Wilcoxon’s test using PHARM/PCS software version 4 (MCS Consulting, Wynnewood, PA, USA). All concentration ranges reported refer to final concentrations of *E. dendroides* extracts and reference compounds in the reaction mixture.

#### 4.5.1. FRAP Assay

This assay was carried out according to Smeriglio et al. [30]. Fifty microliters of sample solution (2.5–100 µg/mL) or Trolox as reference compound (1.25–10 µg/mL) was added to fresh pre-warmed (37 °C) working FRAP reagent (1.5 mL) and incubated for 4 min at RT in the dark. Absorbance was recorded at 593 nm against a blank consisting of the extraction hydroalcoholic mixture 

#### 4.5.2. ORAC Assay

Oxygen radical absorbance capacity was evaluated according to Smeriglio et al. [31]. Briefly, 20 µL of sample solution (1.25–10 µg/mL) diluted in 75 mM phosphate buffer pH 7.4, were added to 120 µL of fresh fluorescein solution (117 nM) and incubated 15 min at 37 °C. After that, 60 µL of 40 mM AAPH solution was added to start the reaction, that was recorded every 30 s for 90 min (λ_ex_ 485; λ_em_ 520) by a fluorescence plate reader (FLUOstar Omega, BMG LABTECH, Ortenberg, Germany). A blank consisting of the extraction hydroalcoholic mixture diluted in phosphate buffer and Trolox as the reference compound (0.25–2.0 µg/mL) were included in each assay. 

#### 4.5.3. BCB Assay

The BCB assay was carried out using a β-carotene emulsion prepared according to Smeriglio et al. [32]. Briefly, 0.28 mL of sample solution (10–80 µg/mL) were added to 7 ml of β-carotene emulsion, whereas an emulsion without β-carotene was used as a negative control. The absorbance was recorded at starting time and during incubation at 50 °C every 20 min for 120 min at 470 nm. BHT was used as a reference compound (0.063–0.5 µg/mL).

#### 4.5.4. TEAC Assay

The Trolox equivalent antioxidant capacity of samples was evaluated according to Monforte et al. [33]. The reaction mixture (4.3 mM K_2_S_2_O_8_ and 1.7 mM ABTS solution, 1:5 *v*/*v*) was incubated for 12–16 h in the dark at RT and diluted just before the analyses until an absorbance of 0.7 ± 0.02 (734 nm). Fifty microliters of each sample (3.0–30 µg/mL) were added to 1 mL of the reagent and incubated at RT for 6 min. The absorbance was recorded at 734 nm against a blank consisting of the extraction hydroalcoholic mixture. Trolox was used as a reference compound (0.63–5.0 µg/mL).

#### 4.5.5. ICA Assay

The iron-chelating activity was evaluated according to Bazzicalupo et al. [34]. Briefly, 50 µL of FeCl_2_·4H_2_O solution (2.0 mM) was added to 100 µL of the sample (0.075–0.60 mg/mL) and incubated at RT for 5 min. After that, 100 µL of 5 mM ferrozine and 3 mL of deionized water were added to the mixture and incubated for 10 min at RT. The absorbance was read at 562 nm against a blank consisting of the extraction hydroalcoholic mixture. EDTA was used as a reference compound (1.50–12.0 μg/mL).

#### 4.5.6. DPPH Assay

The DPPH radical scavenging activity was evaluated according to Smeriglio et al. [32]. Briefly, 37.5 µL of sample solution (5–80 µg/ml) was added to fresh DPPH methanol solution (10^−4^ M), vortex-mixed for 10 s, and incubated in the dark at RT for 20 min. Absorbance was recorded at 517 nm against a blank consisting of the extraction hydroalcoholic mixture. Trolox was used as a reference compound (0.63–5.0 μg/mL).

### 4.6. Anti-Inflammatory Activity

The anti-inflammatory activity of *E. dendroides* extracts was evaluated by two simple in vitro colorimetric enzymatic and non-enzymatic assays. Absorbance was recorded by a multi-well plate reader (Multiskan GO; Thermo Scientific, MA, United States). Results, which represent the average of three independent experiments in triplicate (*n* = 3), were expressed as inhibition percentage (%) of the inflammatory/enzyme activity, calculating the IC_50_ with the respective C.L. at 95% by Litchfield and Wilcoxon’s test using the PHARM/PCS software version 4 (MCS Consulting, Wynnewood, PA, USA). All concentration ranges following reported refer to final concentrations of *E. dendroides* extracts and reference compounds in the reaction mixture.

#### 4.6.1. BSA Denaturation Assay

The ability of samples to inhibit the heat-induced bovine serum albumin denaturation was evaluated according to Saso et al. [35]. Briefly, 100 µL of 0.4 % fatty free BSA solution and 20 µL of PBS pH 5.3 were added into a 96-well plate. Therefore, 80 µL of sample solution (31.25–125 µg/mL) were added to the mixture. The absorbance was recorded at 595 nm at starting time and after incubation for 30 min at 70 °C. A blank consisting of the extraction hydroalcoholic mixture was used as a control. Diclofenac sodium was used as a reference compound (6.25–50 µg/mL).

#### 4.6.2. Protease Inhibition Assay

The protease inhibitory activity of samples was evaluated according to Oyedapo and Famurewa [36]. Briefly, 200 µL of sample solution (31.25–250 µg/mL) were added to the reaction mixture consisting of 12 µL of trypsin (10 μg/mL) and 188 µL of 25 mM Tris-HCl buffer (pH 7.5). After that, 200 µL of 0.8% casein was added and the reaction mixture and incubated for 20 min at 37 °C in a water bath. At the end of the incubation time, 400 µL of perchloric acid was added to stop the reaction. The cloudy suspension was centrifuged at 3500 × g for 10 min and the absorbance of the supernatant was recorded at 280 nm against a blank consisting of the extraction hydroalcoholic mixture. Diclofenac sodium was used as a reference compound (6.25–50 µg/mL).

### 4.7. Brine Shrimp Lethality Assay

In order to investigate the toxicity of the extracts, a brine shrimp lethality assay was carried out according to Caputo et al. [37]. Eggs of *Artemia salina* were purchased from a local pet shop, placed in a hatcher chamber containing seawater, and incubated for 48 h at RT with continuous aeration and illumination. Two hundred microliters of each sample (0.0001 to 10 mg/mL) and K_2_Cr_2_O_7_ as a reference compound (500 µg/mL) diluted in seawater were seeded in a 24-well plate. Ten nauplii per well were added and incubated for 48 h in the same conditions reported above. 

Surviving larvae without abnormal swimming behavior were counted after 24 h and 48 h by a stereomicroscope (SMZ-171 Series, Motic, Seneco s.r.l. – Milano, Italy). One negative control (10 larvae treated with seawater only) were also evaluated. Three independent experiments (*n* = 10) were carried out for each treatment. Lethality was calculated using the following equation:

(1) % Lethality=100−[(slt×100)]/slcs 

where *slt* were the survival larvae treated with extracts or K_2_Cr_2_O_7_, whereas *slcs* were the survival larvae treated with seawater (negative control).

### 4.8. Statistical Analysis

Three independent experiments (*n* = 3 and *n* = 10) were carried out for the in vitro cell-free assays and brine shrimp lethality assay, respectively. Results were expressed as the mean ± standard deviation (S.D.). Data were analyzed by one-way analysis of variance (ANOVA) followed by Tukey’s test and Student-Newman-Keuls Method by SigmaPlot12 (Systat Software, Inc., San Jose, CA, USA). Results were statistically significant for *p* ≤ 0.05.

## 5. Conclusions

In conclusion, this is the first study in which extracts of the aerial parts (flowers, leaves, fruits, and branches) of *E. dendroides* were investigated. Phytochemical analyses showed a high content of polyphenols with chlorogenic acid and rutin as the most representative compounds, respectively for phenolic acids and flavonoids in regards to flowers, leaves, and fruits. On the contrary, chlorogenic acid and isovitexin were found as the most representative compounds for the branch extract. However, beyond the most representative compounds, small differences were found in the phytochemical profile of the extracts under examination, which certainly may contribute to the promising biological activity observed. On average, the flower extract showed the highest antioxidant and anti-inflammatory activity followed by fruits, leaves, and branches. Interestingly, all the extracts showed no toxicity, demonstrating indirectly that the toxicity, generally ascribed to this plant species, is due to the terpene component mainly present in the latex. 

These results, which certainly require further cell-based studies to better investigate the biological properties investigated, show clear preliminary evidence of a possible use of the extracts as powerful antioxidant and anti-inflammatory agents.

## Figures and Tables

**Table 1:** Phytochemical screening of the *E. dendroides* leaf (LE), branch (BE), flower (FE), and fruit (FrE) hydroalcoholic extracts. Results were expressed as the mean ± standard deviation of three independent experiments in triplicate (*n* = 3).

Extract	Total Phenols	Total Flavonoids	Vanillin Index	Proanthocyanidins
	g GAE ^a^/100 g DE ^b^	g QE ^c^/100 g DE	g CE ^d^/100 g DE	g CyE ^e^/100 g DE
LE	55.91 ± 1.89	84.85 ± 1.50	2.08 ± 0.14 ^&^	2.06 ± 0.02 ^&^
BE	26.54 ± 1.25 ^§^	46.93 ± 4.00 ^§^	0.44 ± 0.04 ^§^	1.68 ± 0.08 ^§^
FE	64.75 ± 4.63 *	73.75 ± 7.06 ^°^	0.92 ± 0.05 *	2.23 ± 0.04 *
FrE	48.76 ± 4.25	83.11 ± 3.13	0.67 ± 0.02	3.54 ± 0.02

^a^ GAE, Gallic acid equivalents; ^b^ DE, Dry extract; ^c^ QE, Quercetin equivalents; ^d^ CE, Catechin equivalents; ^e^ CyE, Cyanidin equivalents; * *p* < 0.001 vs. LE, BE and FrE; ^§^ *p* < 0.001 vs. LE and FrE; ^°^ *p* < 0.001 vs. LE and BE; ^&^ *p* < 0.001 vs. FrE.

**Table 2:** Polyphenol profile of *E. dendroides* flower (FE), leaf (LE), fruit (FrE), and branch (BE) extracts. Results were expressed as the mean ± standard deviation of three independent experiments in triplicate (*n* = 3).

*n* ^a^	Compound	RT ^b^	λ_max_ (nm)	[M-H]^-^	MW ^c^	FE	LE	FrE	BE
						g/100 g DE ^d^			
1	Gallic Acid	6.97	232;270	169	170	0.08 ± 0.01 *	0.01 ± 0.00 ^§^	0.04 ± 0.00	0.04 ± 0.00
2	3,4 Dihydroxybenzoic acid	13.08	232	153	154	0.01 ± 0.00	0.01 ± 0.00	0.01 ± 0.00	0.01 ± 0.00
3	*p*-Hydroxybenzoic acid	23.97	232; 260	137	138	0.03 ± 0.00 *	-	0.01 ± 0.00	0.01 ± 0.00
4	Catechin	26.95	279	289	290	-	1.03 ± 0.01	-	-
5	Chlorogenic acid	32.40	290; 320	353	354	13.80 ± 0.58 *	11.66 ± 0.37	11.67 ± 0.42	0.74 ± 0.01 ^°^
6	Caffeic acid	34.02	290; 325	179	180	0.02 ± 0.00 *	0.17 ± 0.01 ^§^	0.01 ± 0.00 ^&^	0.04 ± 0.00
7	Epicatechin	38.23	279	289	290	-	0.01 ± 0.00 ^$^	0.12 ± 0.01	-
8	*p*-Cumaric acid	44.38	280; 300	163	164	0.01 ± 0.00	0.01 ± 0.00	0.02 ± 0.00 ^Ψ^	0.01 ± 0.00
9	Eriodictyol-7-*O*-glucoside	48.31	232; 284	449	450	0.08 ± 0.00 *	0.04 ± 0.00 ^§^	0.05 ± 0.00 ^&^	0.06 ± 0.00
10	*m*-Cumaric acid	50.30	230; 274	163	164	0.01 ± 0.00	0.01 ± 0.00	0.01 ± 0.00	0.01 ± 0.00
11	Verbascoside	53.86	230; 330	623	624	-	0.01 ± 0.00	-	-
12	Isovitexin	54.91	284;328	431	432	0.02 ± 0.00 ^&^	-	-	8.02 ± 0.22
13	Isoquercetin	56.37	262; 356	463	464	-	-	-	0.01 ± 0.00
14	Rutin	57.25	256;356	609	610	2.76 ± 0.11 *	0.65 ± 0.04 ^&^	0.59 ± 0.03 ^&^	0.05 ± 0.00
15	Roifolin	59.97	264; 336	577	578	-	-	0.01 ± 0.00	-
16	Kaempferol-3-*O*-rutinoside	60.95	266; 348	593	594	-	-	0.01 ± 0.00	-
17	Isorhamnetin-3-*O*-rutinoside	62.55	255; 356	623	624	0.41 ± 0.02 *	0.17 ± 0.01 ^§^	0.04 ± 0.00 ^&^	0.01 ± 0.00
18	Luteolin	70.77	268; 350	285	286	-	-	0.01 ± 0.00	-
19	Tiliroside	72.24	266; 314	593	594	-	-	0.01 ± 0.00	-
20	Isorhamnetin	75.71	230; 372	315	316	-	-	-	0.01 ± 0.00
21	Apigenin	75.96	267; 336	269	270	-	-	0.01 ± 0.00	-
	**Total**					**17.23**	**13.78**	**12.62**	**9.02**

^a^ *n*. = peak number based on the elution order; ^b^ RT= retention time; ^c^ MW= Molecular weight; ^d^ DE= dry extract; * p < 0.001 vs. LE, FrE and BE; ^§^ *p* < 0.001 vs. FrE and BE; ^°^ *p* < 0.001 vs. LE and FrE; ^&^ *p* < 0.001 vs. BE; ^$^ *p* < 0.001 vs. FrE; ^Ψ^ *p* < 0.001 vs. FE, LE and BE.

**Table 3:** The antioxidant and free radical-scavenging activity of *E. dendroides* flower (FE), leaf (LE), fruit (FrE), and branch (BE) hydroalcoholic extracts. Results were expressed as the half-inhibitory concentration (IC_50_ μg/mL) with confident limits (C.L.) at 95%.

Assay	FE	LE	FrE	BE	Standard ^a^
	IC_50_ µg/mL (C.L.)				
ORAC	3.68 (1.30–5.46)	6.39 (5.32–7.58)	4.35 (2.15–8.79)	8.39 (6.87–10.25)	0.68 (0.38–1.27) *
TEAC	5.85 (4.90–6.98) ^°^	22.31 (17.31–28.76) ^Ψ^	13.88 (11.46–16.02)	14.17 (11.62–17.28)	2.89 (1.79–3.37) *
ICA	297.97 (147.01–403.96)	201.74 (167.03–243.68)	149.64 (76.74–291.77)	293.66 (247.85–347.93) ^&^	6.62 (5.35–7.89) *
FRAP	10.36 (3.82–28.09) ^$^	42.26 (33.19–53.81)	20.53 (10.35–40.70)	49.95 (13.12–68.38)	3.70 (1.65–6.29) *
DPPH	17.86 (15.75–20.26) ^°^	31.75 (27.25–36.99)	25.72 (21.31–31.05)	30.07 (25.81–35.04)	3.95 (1.39–5.65) *
BCB	12.63 (9.52–16.75) ^&^	24.01 (17.14–33.63)	14.62 (10.87–19.66) ^£^	30.96 (21.16–45.30)	0.24 (0.17–0.32) *

^a^ Trolox for ORAC, TEAC, FRAP and DPPH; Ethylenediaminetetraacetic acid (EDTA) for iron-chelating activity and butylhydroxytoluene (BHT) for β-carotene bleaching; ICA, Iron-chelating activity; BCB, β-carotene bleaching. * *p* < 0.001 vs. all *E. dendroides* extracts; ^°^ *p* < 0.001 vs. LE, FrE, BE; ^Ψ^ *p* < 0.001 vs. FrE and BE; ^&^ *p* < 0.001 vs. LE and FrE; ^$^ *p* < 0.001 vs. LE; ^£^ *p* < 0.001 vs. BE.

## Data Availability

The data presented in this study are available on request from the corresponding author.
